# Pressure-overload-induced angiotensin-mediated early remodeling in mouse heart

**DOI:** 10.1371/journal.pone.0176713

**Published:** 2017-05-02

**Authors:** Jeremy H. Kim, Ya-Ping Jiang, Ira S. Cohen, Richard Z. Lin, Richard T. Mathias

**Affiliations:** Department of Physiology and Biophysics, State University of New York at Stony Brook, Stony Brook, New York, United States of America; Max Delbruck Centrum fur Molekulare Medizin Berlin Buch, GERMANY

## Abstract

Our previous work on angiotensin II-mediated electrical-remodeling in canine left ventricle, in connection with a long history of other studies, suggested the hypothesis: increases in mechanical load induce autocrine secretion of angiotensin II (A2), which coherently regulates a coterie of membrane ion transporters in a manner that increases contractility. However, the relation between load and A2 secretion was correlative. We subsequently showed a similar or identical system was present in murine heart. To investigate whether the relation between mechanical load and A2-mediated electrical remodeling was causal, we employed transverse aortic constriction in mice to subject the left ventricle to pressure overload for short-term (1 to 2 days) or long-term (1 to 2 weeks) periods. Heart-to-body weight ratios and cell capacitance measurements were used to determine hypertrophy. Whole-cell patch clamp recordings of the predominant repolarization currents I_to,fast_ and I_K,slow_ were used to assess electrical remodeling. Hearts or myocytes subjected to long-term load displayed significant hypertrophy, which was not evident in short-term load. However, short-term load induced significant reductions in I_to,fast_ and I_K,slow_. Incubation of these myocytes with the angiotensin II type 1 receptor inhibitor saralasin for 2 hours restored I_to,fast_ and I_K,slow_ to control levels. The number of I_to.fast_ or I_K,slow_ channels did not change with A2 or long-term load, however the hypertrophic increase in membrane area reduced the current densities for both channels. For I_to,fast_ but not I_K,slow_ there was an additional reduction that was reversed by inhibition of angiotensin receptors. These results suggest increased load activates an endogenous renin angiotensin system that initially reduces I_to,fast_ and I_K,slow_ prior to the onset of hypertrophic growth. However, there are functional interactions between electrical and anatomical remodeling. First, hypertrophy tends to reduce all current densities. Second, the hypertrophic program can modify signaling between the angiotensin receptor and target current.

## Introduction

Cardiac myocytes are able to adjust contractility in response to changes in hemodynamic load. The immediate response is through Starling’s law, but subsequently electrical remodeling occurs and finally structural remodeling. With regard to the studies presented here, structural remodeling is defined as a long term increase/decrease in myocyte radii, resulting in an increase/decrease in contractility of individual myocytes. Electrical remodeling is defined as either a long or short term increase/decrease in activity of plasma membrane ion transporters, resulting in an increase/decrease in intracellular calcium and contractility of individual myocytes. There can also be changes in the contractile state of the myofilaments and in calcium handling by the sarcoplasmic reticulum within individual myocytes, and changes to the heart as an organ, but these are not addressed in the current study.

Sustained increases in mechanical strain trigger the cardiac myocyte hypertrophic program, which includes induction of transcription factors as well as the reactivation of fetal genes [1–2]. Re-expressed fetal genes in adult heart cells allow increased protein synthesis, which is associated with increased myocyte radii. In particular, expression levels of myosin heavy chain and α-actinin increase dramatically in hypertrophied cells. Functionally, the synthesis and addition of sarcomeric proteins increases contractility.

**Fig 1:**
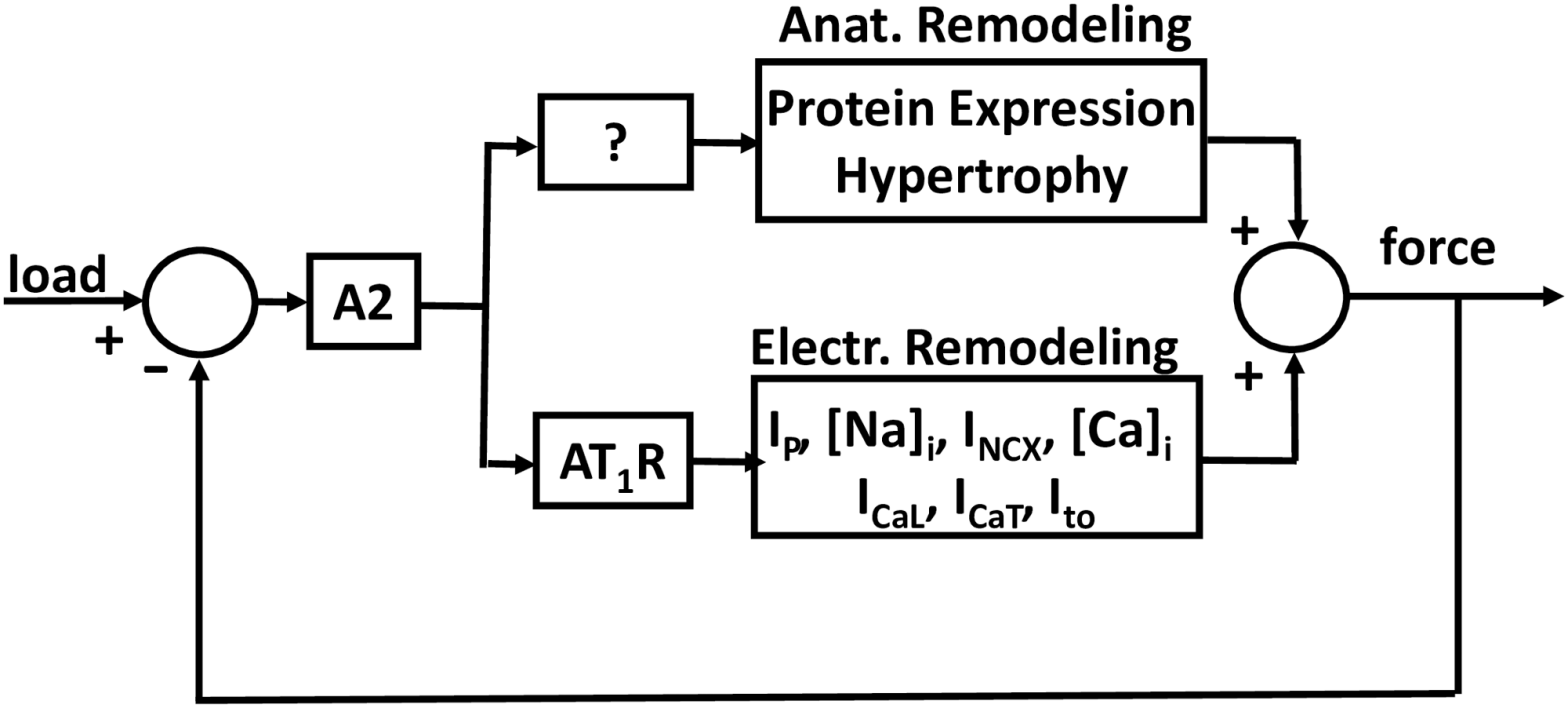
A model of feedback control regulation of contractile force. The currents being regulated include Na/K pump (I_P_), Na/Ca exchange (I_NCX_), L-type calcium (I_CaL_), T-type calcium (I_CaT_), and the transient outward potassium (I_to_). Increases in load are proposed to cause these currents to change in a manner that prolongs action potential duration and increases intracellular sodium and calcium concentrations, resulting in increased contractility.

Studies have correlated activation of a cardiac RAS with induction of the hypertrophic program [3–5]. However, Harada et al [6] found LT-TAC induced hypertrophy in mouse hearts from Angiotensin II Type 1A Receptor-null mice. Perhaps a different splice variant of the receptor mediates the hypertrophic response, or perhaps A2 induces hypertrophy without receptor activation, but certainly these findings suggest a lack of understanding of the role of A2 in hypertrophy. In Fig 1, this is indicated by the question mark in the block leading to anatomical remodeling.

With regard to electrical remodeling, Yasuno et al [7] provided data consistent with LT-TAC-induced electrical remodeling being blocked in the angiotensin II type 1A receptor-null mice. Moreover, data included here show LT-TAC-induced electrical-remodeling is eliminated when the AT_1_Rs are inhibited. Other studies have correlated mechanical stress with electrical remodeling [8]. For instance, in the dog left ventricle, rapid pacing caused increased circumferential strain and triggered action potential (AP) prolongations in the same pattern as strain [9]. Moreover, Ozgen et al. [10] showed the effects of rapid pacing involved activation of AT_1_Rs. Activation of AT_1_Rs in canine left ventricle has been shown to reduce the Na/K pump current (I_P_) [11], reduce the transient outward K^+^-current (I_to_) [11–13], and increase L-Type calcium current (I_CaL_) [11,14–16]. All of these effects of AT_1_R activation on cardiac plasma membrane transporters are expected to increase action potential duration, increase calcium entry and increase contractility [11].

Signaling by AT_1_Rs occurs in two stages. Initial activation involves binding of A2 to the extracellular domain of the receptor, resulting in intracellular activation of both G_q_, which activates protein kinase C (PKC), and G_i_, which inhibits protein kinase A (PKA) and phosphoinositide 3 kinase (PI3K). Chronic activation of G_q_ may be involved in the development of cardiac hypertrophy [17,18]. In the second stage of signaling, G protein activation ceases, the receptors associate with β-arrestins and the complex is internalized to form a new signal transduction scaffold [19]. This stage of receptor signaling appears to be involved in the development of electrical remodeling [10–12].

Previous studies have demonstrated local A2 production in cardiac tissue [20]. Moreover, mechanical stretch of isolated cardiac myocytes caused autocrine secretion of A2 and increased activation of AT_1_Rs [21]. Work by Gao et al. [11] on A2-induced electrical-remodeling was performed on acutely isolated myocytes from the canine left ventricle, where they found a transmural gradient in autocrine secretion of endogenous A2 into the T-system lumen. This gradient correlated with the transmural gradient in strain, Endo > Epi [22]. Their data suggested autocrine secretion of A2 generated transmural gradients in I_P_ (Endo < Epi), I_to_ (Endo < Epi) and I_CaL_ (Endo > Epi). Each gradient would affect intracellular calcium and contractility (Endo > Epi).

Based on the above data, Gao et al. [11] hypothesized a feedback control system similar to that shown in Fig 1. The filling pressure of the ventricle (load) is sensed and compared to contractile force production. If load exceeds force production, the difference drives an increase in secretion of A2 into the T-system lumen, where it activates T-system membrane AT_1_Rs. Electrical remodeling is initiated by receptor internalization, which occurs in a time frame of about 30 minutes. The path to anatomical remodeling may also involve A2 secretion, as reviewed above, but since hypertrophy occurs in AT_1_R-null mice, it is not fully understood, so we include an unknown step between A2 and hypertrophy in Fig 1. Regardless of the signaling pathway, hypertrophy requires a signal to the nucleus, where protein synthesis is initiated, which requires several days. The two pathways converge, as activation of either increases force production. However, because of the time difference between activation of the two pathways, we hypothesized that anatomical remodeling will only occur if electrical remodeling is insufficient to restore balance between load and force.

The hypotheses in Fig 1 were primarily based on data from the canine heart, however results presented in Kim et al. [12] suggested a similar autocrine renin angiotensin system (RAS) exists in left ventricular myocytes from the mouse heart. We have therefore used the mouse in the current study to further test the hypotheses in Fig 1. Aortic banding was used to increase the filling pressure of the left ventricle over short term (ST 1–2 days) or long term (LT 1–2 weeks) periods. Acutely isolated myocytes from the left ventricle of these mice were compared to control myocytes to determine if the increased load led to electrical remodeling, if electrical remodeling preceded anatomical remodeling, and if there were interactions between electrical and anatomical remodeling.

## Materials and methods

### Transverse aortic constriction

In 2 to 3 month old wild-type C57/BL6 mice, transverse aortic constriction (TAC) was employed to apply pressure-overload-induced mechanical strain on left ventricular (LV) myocytes. The State University of New York at Stony Brook’s Institutional Animal Care and Use Committee (IACUC) approved this research. The approved IACUC number is 2012–1712. The protocol for the surgical procedure used in these studies is outlined in a previous report [23]. A 27-gauge needle was used for ligation, which induced hypertrophy within 1 week of surgery. For each TAC mouse, the same surgery without aortic constriction (SHAM) was performed to serve as a negative control. Identical procedures, with exception to ligation, were followed for both TAC and SHAM surgery. TAC and SHAM mice were sacrificed at 1 to 2 days (short-term) or 1 to 2 weeks (long-term) post-surgery to examine the effects of short-term (ST) and long-term (LT) aortic constriction on isolated cardiac myocytes.

### Isolation of left ventricular myocytes

At the appropriate time points following TAC or SHAM surgery, mice were euthanized via CO_2_ inhalation in an enclosed chamber. Immediately following euthanasia, the weight of each animal was recorded. The chest cavity was opened and the heart was excised and immediately transferred into a 35 mm dish with ice-cold Normal Tyrode Solution containing (in mM) 137.7 NaCl, 2.3 NaOH, 5.4 KCl, 1 MgCl_2_, 10 Glucose and 5 HEPES (pH adjusted to 7.4 with NaOH). Extraneous tissues surrounding the aorta including the lungs, thymus and adipose tissue were carefully cleaned off using small scissors to expose the aorta for cannulation. The heart was then weighed in order to calculate the heart to body weight ratio for each mouse. The heart weight included both atria, both ventricles, part of the aorta and blood/Tyrode's left over in the chambers and vessels. This procedure was used because the heart was subsequently dissociated into ventricular myocytes for whole cell patch clamp studies. In studies by others, the atria and aorta were removed and the heart dried prior to weighing. This gave a normal HW/BW of 4–6 mg/g, depending on age and genetic background, whereas our procedure gives a normal HW/BW of about 8 mg/g. Our comparison of HW/BW in sham vs TAC hearts was done on mice of the same genetic background, of about the same age, that had been housed in the same place and fed the same chow and were subjected to the same surgical procedure, except the TAC was not applied in sham hearts.

Ventricular myocytes were isolated as described in Kim et al. [12]. Briefly, the heart was cannulated and perfused using a temperature-controlled Langendorf apparatus. Perfusion with a Normal Tyrode Solution containing 0.16 mg/ml Liberase TH (Roche Applied Science Inc., Indianapolis, IN) was used for tissue digestion. After washing, the heart was placed in a dish containing KB solution (in mM) 83 KCl, 30 K_2_HPO_4_, 5 MgSO_4_, 5 Na-Pyruvic Acid, 5 β-OH-Butyric Acid, 5 Creatine, 20 Taurine, 10 Glucose, 0.5 EGTA and 5 HEPES (pH adjusted to 7.2 with HCl) and the left ventricle was separated from the rest of the heart. The left ventricle was gently teased apart by mechanical agitation and filtered through a nylon mesh to collect the cell suspension. The isolated myocytes were stored in KB solution at 22°C.

### Cell preparation

Cells obtained from both SHAM and TAC mice were divided into treated and untreated groups, where cells in the treated group were incubated at 22°C for at least 2 hours in KB solution with 5 μM of the AT_1_R blocker saralasin (Sigma-Aldrich Inc.). Because of reports suggesting an agonistic property of saralacin, in early studies we used losartan as the AT_1_R blocker [13]. However, when we compared the effects of the two inhibitors, either inhibitor completely reversed electrical remodeling induced by exogenous A2, and completely inhibited electrical remodeling induced by endogenous A2. We therefore saw no “agonistic” effects of saralacin and have continued to use it as our inhibitor of choice.

### Electrophysiological recordings

Whole-cell patch clamp experiments were conducted at room temperature (22°C) within 12 hours of cell isolation. For K^+^-current measurements, the internal pipette solution contained (in mM) 115 K-Aspartic Acid, 25 KOH, 10 KCl, 3 MgCl_2_, 11 EGTA, 10 HEPES and 5 Na_2_-ATP (pH adjusted to 7.2 with KOH). Pipette series resistances were between 4–7 MΩ in whole-cell mode. During patch clamp recordings, myocytes were perfused with an external solution containing (in mM) 137.7 NaCl, 2.3 NaOH, 5.4 KCl, 1 MgCl_2_, 1.8 CaCl_2_, 2 CoCl_2_, 10 Glucose and 5 HEPES (pH adjusted to 7.4 with NaOH). Voltage-clamp experiments were performed using an Axopatch 1D amplifier (Axon Instruments Inc.) interfaced to a computer with a Digidata 1200 digitizer and pClamp 8.2 software (Axon Instruments Inc.). K^+^-currents were measured in response to a short prepulse (10 ms) to −30 mV to inactivate Na^+^ channels followed by 6000 ms voltage steps (V_test_) between +10 mV and +50 mV from a holding potential of −65 mV. For all voltage-clamp recordings, series resistance was compensated electronically by ~85%. Cell capacitance (C_m_) was measured from each cell using the Membrane Test function in the pClamp software package.

### Data analysis and statistics

Voltage-clamp data were analyzed using Clampfit 8.2 (Axon Instruments Inc.), Microsoft Excel (Microsoft Inc.), SigmaPlot (Systat Software Inc.) and MATLAB (Mathworks Inc.). Distinct K^+^ current components (I_to,fast_, I_K,slow_ and I_sus_) were extracted from the overall K^+^ current recordings using the Curve Fitting Toolbox in MATLAB to fit a two-exponential decay function in the form of I(t) = A_1_exp(-t/τ_1_) + A_2_exp(-t/τ_2_) + A_3_ [12, 24]. A_1_ and A_2_ represent peak amplitudes of I_to,fast_ and I_K,slow_. Their respective inactivation time constants differed by a factor of approximately 20. I_to,fast_ was identified as the inactivating component with a decay time constant less than 100 ms and I_K,slow_ was identified as the slower inactivating component (τ_slow_ > 1000 ms). A_3_ represents the steady-state current I_sus_. Current amplitudes were normalized to C_m_ and presented as current densities (pA/pF). Assuming the specific membrane capacitance is about 1 μA/cm^2^, 1 pA/pF ≈ 1 μA/cm^2^.

Pooled results are expressed as mean ±SE. Statistical differences in cell capacitance or heart weight between TAC and SHAM groups were assessed with the student’s t-test. In either long term or short term studies of K^+^-currents, there are 4 conditions: banded or not banded, and saralasin or no saralasin. One-way analysis of variance (ANOVA) was used to determine whether there were statistical differences between these four current-voltage relationships in either long term or short term experiments. Current-voltage relationships for either I_to,fast_ or I_K,slow_ converge to zero current at the reversal voltage, presumably near E_K_. There can be no statistical difference between the currents at the reversal potential and statistical differences disappear as the reversal voltage is approached. For this reason, we have chosen to evaluate statistical differences between multiple comparisons of the currents at a voltage of +50 mV, which is as far from the reversal potential as we recorded. The acid tests of these differences were *post hoc* comparisons between each current using Least Squares Determination (LSD). Significance was defined as P < 0.05.

## Results

### Time dependence of structural remodeling

**Fig 2:**
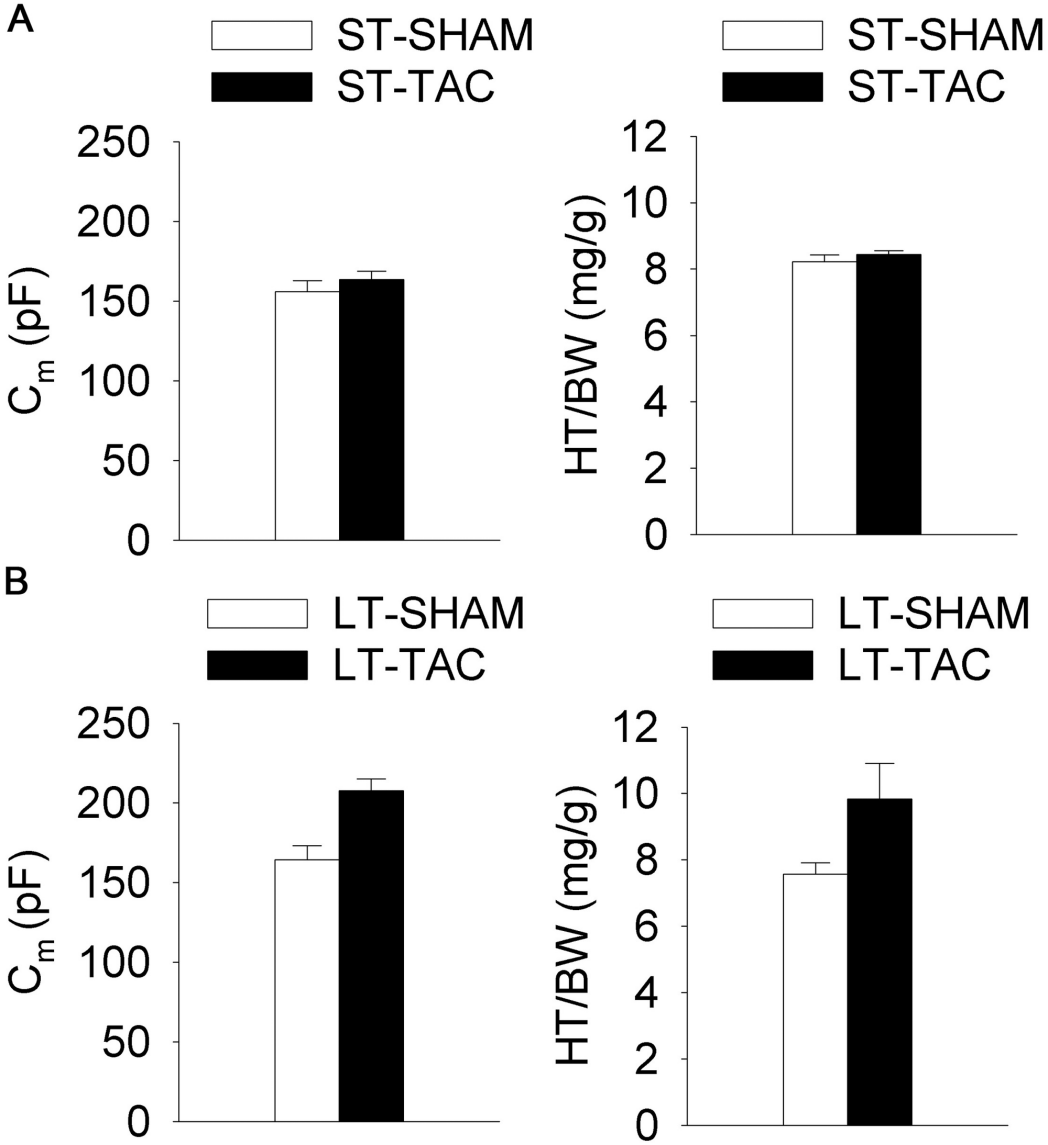
The effect of increased load on cell capacitance (C_m_) and heart-to-body weight ratios (HT/BW). **A.** Measurements of C_m_ and HT/BW from ST-SHAM and ST-TAC mice. There were no significant differences between these two groups. **B.** Measurements of C_m_ and HT/BW from LT-TAC and LT-SHAM mice. LT-TAC induced a significant increases in myocyte size and overall heart mass (P < 0.05). Thus hypertrophy was not detected at up to 2 days post load increase, but was clearly detected by 1 week post load increase. Compared to studies of electrical remodeling, the results presented here show hypertrophy is relatively slow to develop.

To determine the extent of structural remodeling, cell capacitance (C_m_) was used as a measure of cell size, and heart to body weight ratio (HT/BW) was used as a measure of heart size. Myocytes subjected to surgically induced transvers aortic constriction (TAC) were compared control myocytes (SHAM) subjected to surgery without TAC. As illustrated in Fig 2A, C_m_ values in short-term TAC (ST-TAC) and SHAM (ST-SHAM) myocytes were not significantly different (ST-SHAM: 156 ± 7 pF, 14 cells; ST-TAC: 164 ± 5 pF, 15 cells). Similarly, HT/BW measurements were not significantly different in these two groups (ST-SHAM: 8.22 ± 0.21 mg/g, 5 mice; ST-TAC: 8.44 ± 0.12 mg/g, 5 mice). In contrast, relative to SHAM controls, myocytes from hearts subjected to long-term aortic constriction (LT-TAC) were characterized by significant increases in both C_m_ (LT-SHAM: 164 ± 9 pF, 12 cells; LT-TAC: 208 ± 7 pF, 14 cells; 27% increase) and HT/BW (LT-SHAM: 7.57 ± 0.34 mg/g, 6 mice; LT-TAC: 9.83 ± 1.08 mg/g, 6 mice; 30% increase) as shown in Fig 2B.

These studies found no evidence of hypertrophy in hearts subjected to 1 to 2 days of pressure overload. However, hearts subjected to 1 to 2 weeks of pressure overload showed clear signs of hypertrophy. Myocytes isolated from these hearts had significantly increased size, as indicated by an increased cell capacitance compared to controls, and the hearts themselves had a significantly larger mass than controls, as indicated by an increased heart to body weight. The capacitance and HT/BW of TAC myocytes both increased by a factor of about 1.3.

### I_to,fast_ and I_K,slow_ remodeling in ST-TAC myocytes

We examined the short term effect of increased load on I_to,fast_ and I_K,slow_, which contribute significantly to AP repolarization. Reductions in these currents prolong the AP [12], increase calcium entry, and increase contractility. The hypothesis in Fig 1 is that a short-term (ST) increase in load (TAC) will induce autocrine secretion of A2, activation of AT_1_Rs and inhibition of these currents. Each type of current was studied in four conditions. ST-SHAM was the control condition of surgery without TAC, where the surgery was performed 1–2 days before cell isolation and experiments. In ST-TAC, the hearts were subjected to pressure overload for a 1–2 day period, then cells were isolated for experiments. Saralacin (Sar) is a specific inhibitor of the AT_1_R, so if the A2 hypothesis is correct, the effect of ST-TAC on these currents should be reversed by application of Sar. As another control, Sar was applied to ST-SHAM myocytes, where it was expected to produce relatively smaller reductions in the currents, depending on basal AT_1_R activation.

**Fig 3:**
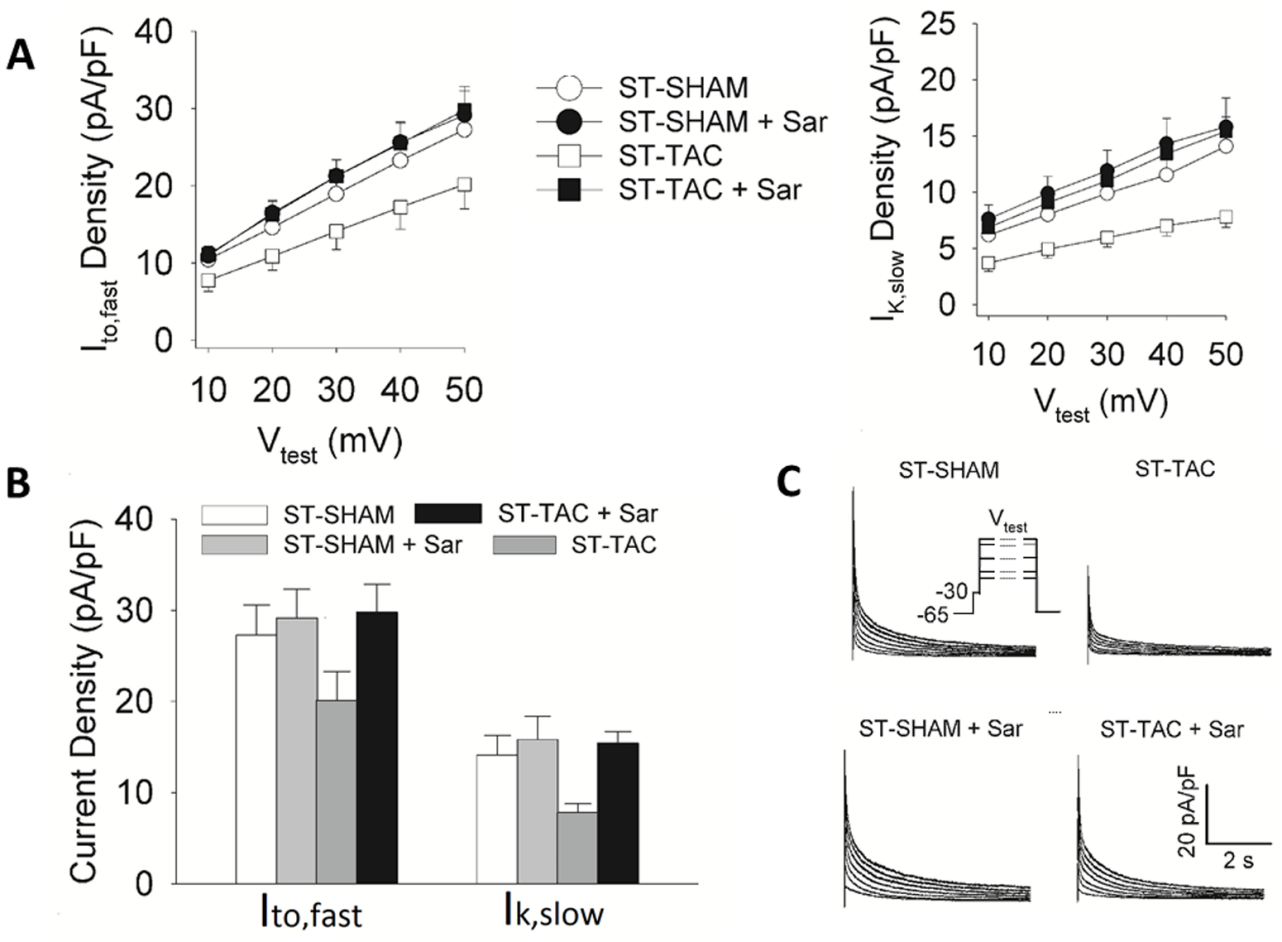
The effects of ST-TAC on I_to,fast_ and I_K,slow_ current densities. **A.** Voltage dependences of peak I_to,fast_ and I_K,slow_ densities obtained from ST-SHAM and ST-TAC myocytes in the presence and absence of Sar. **B.** I_to,fast_ and I_K,slow_ peak current densities at V_test_ = +50 mV. Either I_to,fast_ or I_K,slow_ was significantly reduced in ST-TAC myocytes relative to the other three conditions (P < 0.05). Neither I_to,fast_ nor I_K,slow_ was significantly altered by the other three conditions. These data suggest increased load activates an autocrine RAS that initiates electrical remodeling well in advance of the hypertrophic response. **C.** Representative whole-cell patch clamp traces of outward K^+^-currents from ST-SHAM and ST-TAC myocytes in the presence and absence of the AT_1_R blocker saralasin (Sar). Inset on the left side depicts the voltage clamp protocol used to obtain the current traces (V_test_: test potential).

The peak current-voltage (I-V) relationships of I_to,fast_ and I_K,slow_ are shown in Fig 3A. Peak currents at +50 mV in the 4 experimental conditions for either I_to,fast_ or I_K,slow_ are shown in Fig 3B. These were compared using one way ANOVA, which indicated the four current densities were not all the same for either I_to,fast_ or I_K,slow_. Post hoc multiple comparisons using LSD showed both I_to,fast_ and I_K,slow_ were significantly lower in ST-TAC than in the other 3 conditions, whereas the values of current in the other 3 conditions were not statistically different from each other. Typical traces of outward K^+^ currents are shown in Fig 3C.

As hypothesized in Fig 1, these data suggest increased load induces autocrine A2 secretion and AT_1_R activation, which in turn induces reductions in repolarizing K^+^-currents. I_to,fast_ was reduced by a factor of about 1.4 (SHAM/TAC) and I_K,slow_ was reduced by a factor of about 1.8. This causes prolongation of the AP [12], so presumably calcium entry and contractility have increased in response to the increase in load.

The hypothesis for Fig 1 is electrical remodeling is the initial response to changes in load, but its effect is limited, so if the increase in load is too great to be negated by electrical remodeling the hypertrophic response follows. We have shown the hypertrophic response is detectable after one week of pressure overload (Fig 2), but we do not know if this response replaces or supplements short-term electrical remodeling.

### The effect of LT-TAC on current densities

Based on the data shown in Fig 2, LT-TAC induced hypertrophy, which increased myocyte capacitance by a factor of about 1.3. To estimate current densities, we divide the current amplitudes by cell capacitance. If there is no change in channel synthesis and expression, the current density in LT-TAC in the absence of AT_1_R-induced electrical remodeling (LT-TAC+Sar) should be a factor of about 1.3 lower than the current density in LT-SHAM+Sar. If electrical remodeling is induced, then the current density in LT-TAC should be significantly lower than in LT-TAC+Sar. The current density in LT-TAC+Sar should be intermediate between LT-SHAM (or LT-SHAM+Sar) and LT-TAC. If no electrical remodeling is present in the LT-TAC myocytes, and there is no change in channel synthesis and expression, then Sar should have only small effects; the current density of LT-TAC should be a factor of about 1.3 lower than LT-SHAM, and the current density in LT-TAC+Sar should a factor of about 1.3 lower than that in LT-SHAM+Sar.

**Fig 4:**
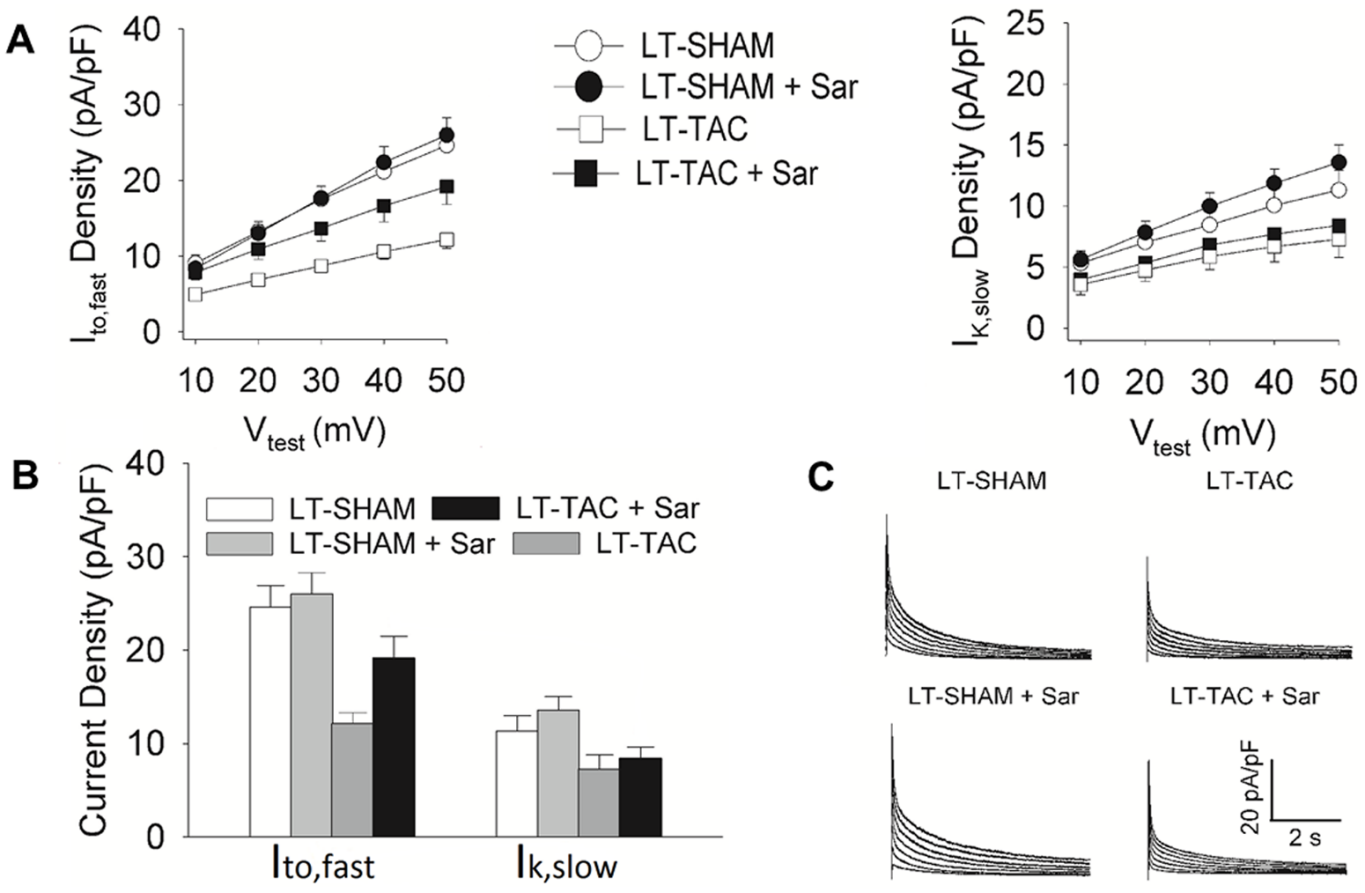
The effects of LT-TAC on I_to,fast_ and I_K,slow_ current densities. **A.** Voltage dependences of peak I_to,fast_ and I_K,slow_ densities measured from LT-SHAM and LT-TAC myocytes in the presence and absence of Sar. **B.** I_to,fast_ and I_K,slow_ peak current densities at V_test_ = +50 mV. I_to,fast_ was significantly reduced in LT-TAC relative to the other three conditions (P < 0.05). I_to,fast_ in LT-SHAM and LT-SHAM+Sar myocytes was not significantly different, however I_to,fast_ in LT-TAC+Sar was significantly reduced relative to LT-SHAM+Sar (P < 0.05). I_K,slow_ in LT-SHAM and LT-SHAM+Sar were not significantly different. Similarly, I_K,slow_ in LT-TAC and LT-TAC+Sar were not significantly different. However, I_K,slow_ in either LT-TAC verse LT-SHAM or LT-TAC+Sar verse LT-SHAM+Sar was significantly reduced (P < 0.05). Possible reasons for these patterns of change are discussed in the text. **C.** Representative traces of K^+^-currents from LT-SHAM and LT-TAC myocytes in the presence and absence of the AT_1_R blocker saralacin (Sar).

The I-V relationships for I_to,fast_ and I_K,slow_ in each of the 4 conditions studied are shown in Fig 4A. Fig 4B compares peak current densities at +50 mV for each of the 4 conditions studied. The time courses of typical K^+^-current densities for 2 control (SHAM) myocytes and 2 LT-TAC myocytes are shown in Fig 4C.

#### I_to,fast_

One way ANOVA indicated the 4 current densities in Fig 4B were not all the same. *Post hoc* LSD multiple comparisons of the 4 current densities were performed. The current density in LT-TAC (12 pA/pF) was significantly reduced in comparison to any of the other 3 conditions, suggesting a load-dependent AT_1_R-mediated reduction I_to,fast_ is present. The current densities in LT-SHAM vs LT-SHAM+Sar were not significantly different, indicating low basal activity of AT_1_Rs, consistent with the ST-SHAM data in Fig 3. The current density in LT-TAC+Sar (19 pA/pF) was significantly lower than in LT-SHAM+Sar (26 pA/pF), with the ratio being about 1.4, which is close to the previously measured average increase in C_m_. The current density in LT-TAC was reduced by a factor of about 1.6 in comparison to that in LT-TAC+Sar, comparable to the reduction in ST-TAC of a factor of about 1.5. It appears the reduction in I_to,fast_ initiated by ST-TAC has persisted without significant change in the LT-TAC myocytes. The overall reduction in current density by a factor of 2.0 in LT-TAC in comparison to LT-SHAM depends on both the increase in C_m_, which is not reversible in the short-term, and the decrease in the number of open channels, which can be reversed in the short-term by inhibition of AT_1_Rs.

These results suggest a short-term increase in load induced persistent AT_1_R-mediated reversible reduction in I_to,fast_, but electrical remodeling was insufficient to offset the large increase in load, so structural remodeling ensued. The increase in membrane area associated with hypertrophy caused the I_to,fast_ current density to further decrease, which was not reversible in the short-term. A different pattern was seen for I_K,slow_.

#### I_K,slow_

One way ANOVA indicated the 4 current densities in Fig 4B were not all the same. *Post hoc* LSD multiple comparisons of the 4 current densities were performed. The current densities in LT-SHAM (11 pA/pF) and LT-SHAM+Sar (13 pA/pF) were not statistically different. Similarly, the densities in LT-TAC (7 pA/pF) and LT-TAC+Sar (8 pA/pF) were not significantly different. Thus, there was little if any reversible AT_1_R-mediated reduction in the number of open channels. The current density in LT-TAC was decreased relative to LT-SHAM, however the reduction was by a factor of about 1.6, which is similar to the increase in C_m_. Similarly, the current density in LT-TAC+Sar was significantly decreased relative to LT-SHAM+Sar, however the reduction was again by a factor of about 1.6, which is again similar to the increase in C_m_.

These data suggest the AT_1_R-mediated reduction in the number of open I_K,slow_ channels induced by ST-TAC has disappeared in the LT-TAC myocytes. This is most clearly demonstrated by the observation that Sar has no significant effect on LT-TAC current densities. The LT-TAC induced reductions in current densities were most likely due to the increase in C_m_ associated with hypertrophy.

### The effect of LT-TAC on total current

**Fig 5:**
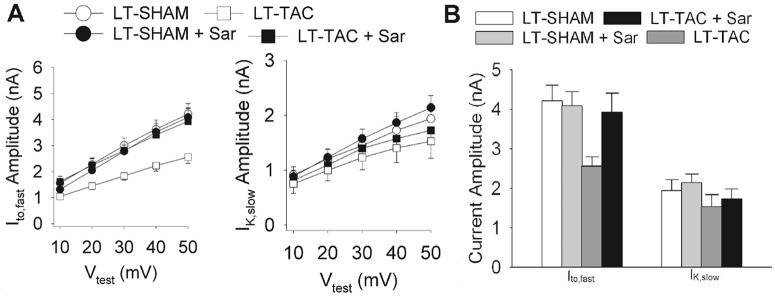
The effects of LT-TAC on I_to,fast_ and I_K,slow_ total currents. **A.** Voltage dependences of peak I_to,fast_ and I_K,slow_ amplitudes measured from LT-SHAM and LT-TAC myocytes in the presence and absence of the AT_1_R blocker saralacin (Sar). **B.** I_to,fast_ and I_K,slow_ peak current amplitudes at V_test_ = +50 mV. I_to,fast_ was significantly reduced in LT-TAC myocytes relative to the other three conditions (P < 0.05). There was no significant difference in I_to,fast_ in the other three conditions. There was no significant difference in I_K,slow_ in any of the four conditions. These data suggest there was no change in the number of either I_to,fast_ or I_K,slow_ channels. However, I_to,fast_ remains coupled to ST changes in AT_1_R activation, whereas I_K,slow_ is uncoupled from the receptor by the hypertrophic program.

In the previously described experiments, total current was divided by total capacitance to obtain a current density (pA/pF). In control conditions, cell capacitance varied from around 140 pF to 215 pF, so this is a significant source of variability in the measurement of total current, and this variability was removed by the normalization. However, average capacitance is increased in hypertrophy and this complicated the comparison of current densities in the previous figure. Un-normalized total currents are compared in Fig 5. The I-V relationships for total I_to,fast_ and I_K,slow_ in each of the 4 conditions studied are shown in Fig 5A. Fig 5B compares peak current at +50 mV for each of the 4 conditions studied.

#### I_to,fast_

One way ANOVA of the peak currents shown in Fig 5B did not quite reach statistical significance (P = 0.055). However, *post hoc* LSD multiple comparisons of the 4 peak currents found the total current in LT-TAC myocytes (2.4 μA) was significantly reduced in comparison to any of the other 3 currents, whereas the other 3 currents were all about 4 μA and were not statistically different. The reduction of current in LT-TAC myocytes in comparison to LT-TAC+Sar (3.9 μA) was a factor of about 1.6, which is approximately the same as the previous measurement of the reduction in current density (~1.5).

These data suggest the total number of I_to,fast_ channels did not significantly change in LT-TAC, but the total number of open I_to,fast_ channels was reduced. The reduction, however, was through short-term reversible activation of AT_1_Rs. These results are consistent with the conclusions presented in the text describing I_to,fast_ in the last figure.

#### I_K,slow_

One way ANOVA of the 4 peak currents presented in Fig 5B showed no significant difference. *Post hoc* LSD multiple comparisons also showed no significant difference between any of the currents, which were all around 1.7 nA.

These data suggest no change in the total number of I_K,slow_ channels in LT-TAC. Moreover, the AT_1_R-mediated reduction in I_K,slow_ seen in ST-TAC disappears in LT-TAC. Nevertheless, activation of AT_1_Rs persists in LT-TAC, since I_to,fast_ retains its sensitivity to AT_1_R inhibition. Apparently the receptor is uncoupled from I_K,slow_ by the hypertrophic program. These results are consistent with the conclusions presented in the text describing I_K,slow_ in the last figure.

## Discussion

We employed aortic constriction in mice to investigate the time dependence and functional relationship of electrical and mechanical remodeling in the heart. Additionally, we investigated the role of AT_1_Rs in mediating changes in I_to,fast_ and I_K,slow_ induced by mechanical stress. The working hypothesis for this study was that increased mechanical load to the left ventricle induces autocrine secretion of A2 by myocytes into the T-system lumen, where it activates AT_1_Rs, which coherently affect a coterie of ion transporters [11]. Based on a number of studies discussed in Gao et al. [11], each of the A2-induced changes in transport should lead to increased contractility. We did not study all of the transporters involved. Instead we used decreases in the dominant repolarizing currents, I_to,fast_ and I_K,slow_, as markers of changes in transport that are expected to increase contractility.

### Short-term aortic constriction

Aortic constriction for 1 to 2 days was not sufficiently long to induce hypertrophy in ventricular myocytes, but both I_to,fast_ and I_K,slow_ were significantly reduced through activation of AT_1_Rs. A previous study [12] showed exogenous A2 caused AP prolongation and inhibited I_to,fast_ and I_K,slow_, presumably through the same path as ST-TAC-induced electrical remodeling. Indeed, the inhibitions due to saturating [A2] were the same as the inhibitions due to ST-TAC. Both studies are consistent with the hypothesis that load-induced autocrine secretion of A2 leads to electrical remodeling and increased contractility [11].

### Long-term aortic constriction

In the time it takes for hypertrophy to take place, there could also be changes in synthesis and expression of channel forming proteins and signaling proteins. We therefore had no specific hypothesis for the effect on electrical remodeling. However, since the hypertrophic program leads to increased contractility, we expected the electrical changes would be in the direction to increase contractility.

Aortic banding for 1 to 2 weeks induced significant hypertrophy of the heart and individual myocytes. In the hypertrophied myocytes, total I_to,fast_ (pA) was reduced by a factor of about 1.6, which is essentially the same as seen in short time studies. Moreover, as seen in short-term studies, inhibition of AT_1_Rs restored the total current to control levels, implying no change in the number of channel forming proteins. However in hypertrophied myocytes, the current density for I_to,fast_ (pA/pF) was additionally reduced by the increase in membrane area. Thus the current density was reduced more than seen in short time studies.

The pattern of change for I_K,slow_ was different. In hypertrophied myocytes, total I_K,slow_ (pA) was the same as in control myocytes but was insensitive to AT_1_R inhibition. Thus there was no change in the number of channel forming proteins, but the coupling of I_K,slow_ to short-term changes in AT_1_R activity disappeared with the advent of hypertrophy. Nevertheless, there was still a reduction in current density for I_K,slow_ (pA/pF) due to the hypertrophic membrane area increase.

The effect of LT-TAC on total current differed from its effect on current density, so which matters? With regard to the effect on the AP, the time course of voltage changes depends on the membrane rate constant (conductance/capacitance), so current density is the relevant factor. But the increase in membrane area due to hypertrophy will tend to reduce current densities of all membrane transporters. Decreases in the L-type calcium current density, T-type calcium current density or persistent sodium current density would shorten the AP, reduce calcium entry and reduce contractility. Since the purpose of the hypertrophic program is to increase contractility, one expects there are long-term changes in these currents to make them consistent with this program. The effect of LT-TAC on the action potential and contractility cannot, therefore, be understood without measuring the effect of LT-TAC on all membrane transporters.

The above point is particularly relevant because the present study has shown the effect of LT-TAC on electrical remodeling is transporter specific. In LT-TAC, the coupling of short-term changes in AT_1_R activity to I_K,slow_ disappears whereas it remains unchanged for I_to,fast_. Why did I_K,slow_ lose its connection to AT_1_R activation? One possibility to be considered is that electrical remodeling has the potential to be arrhythmogenic [7]. Perhaps LT-TAC induced reductions in repolarizing current density were approaching their arrhythmogenic limit.

In summary, long-term increases in load cause persistent AT_1_R activation, probably through load-induced autocrine secretion of A2. The result is electrical remodeling, which leads to increased contractility. Long term increases in load also induce the hypertrophic program, which also leads to increased contractility. However, the relationship of the hypertrophic program to the autocrine RAS is not well understood [6]. Moreover, the hypertrophic program feeds back into the program for electrical remodeling, in a manner, we speculate, that produces a coherent overall response that is not arrhythmogenic but maximizes contractility.

### Comparison with previous studies

Previous studies using aortic constriction in mice reported significant hypertrophy at various post-operation durations, which ranged from 7 days [25] to 3 weeks or more [26]. Consistent with these studies, we observed that 1 week was sufficient to induce hypertrophy using similarly sized needles for ligation. A separate report [27] documented changes in LV weight to body weight ratio from one hour to 48 hours post-operation. In the initial 48 hours, no significant change was detected, consistent with our findings.

In the current study, we did not measure AP duration, however in our previous study [12] we showed 5 μM A2 increased duration by a factor of about 1.6. The effect of either ST-TAC or 5 μM exogenous A2 on the repolarizing current I_to,fast_ was a reduction by a factor of about 1.5, and for I_K,slow_ either caused a reduction of about 1.6. Based on the hypothesis that ST-TAC leads to electrical remodeling through autocrine secretion of A2, we expect that ST-TAC caused an increase in duration of around 1.6. With regard to LT-TAC, there is ample time for protein synthesis/degradation, so there is no reason to expect the changes in transport to be the same as in ST-TAC. For example, the increase in membrane area with hypertrophy will reduce the current density generated by all membrane transporters, unless there are further changes in transport activity with LT-TAC following those instituted by ST-TAC. However, Zengyi et al [28] recorded AP duration after 3 weeks of TAC and found it had increased by a factor of about 1.5. AP duration is probably not a very sensitive measure of the changes in underlying currents. These need to be measured.

One concern was that stresses associated with anesthesia and surgery might induce changes at early time points, changes that were independent of aortic banding. However, Spruill, et al. [29] suggested the non-specific effects of stress were minimal by 24 hours in mice with aortic constriction surgery. Moreover, I_to,fast_ and I_K,slow_ in sham-operated controls were essentially the same in ST and LT myocytes, suggesting no short-term effects of surgery.

In previous studies, mechanical strain and AT_1_R activation have been related with two distinct molecular mechanisms. In one study, mechanical strain of cardiac myocytes *in vitro* induced autocrine secretion of A2 and the induction of the hypertrophic program [21]. A more recent study suggested AT_1_R stimulation occurs in response to mechanical stress without the involvement of A2 [30]. However, previous studies [11,12] show that application of exogenous A2 mimics the effects of pressure overload on short-term electrical remodeling. Moreover, the remodeling is reversed by saralasin, a competitive inhibitor of A2 binding to AT_1_Rs, and it is not clear how saralasin would reverse direct mechanical activation of the receptors. Overall the data are more consistent with the Sadoshima et al. [21] model.

### The coupling of A2 with target transport proteins

The first step is binding of extracellular A2 to AT_1_Rs. Gao et al [11] in canine ventricle, then again in Kim et al [12] in mouse ventricle recorded dose inhibition curves for A2 binding. The curves were very similar and the data suggested an elegant system was in place. The K_0.5_s in dog and mouse were ~ 0.2 μM and 0.4 μM respectively, which are similar and much larger than systemic concentrations of A2. So the cardiac receptors have been modified in a manner that ensures they will not respond to systemic A2, but will respond only to the much larger concentrations of local A2 in the heart. Gao et al [11] examined how such high concentrations of A2 were possible in the heart and found the autocrine RAS was exclusively localized to the T-system of myocytes. Since the volume of T-system lumen is very small, a relatively low rate of secretion generated a large transmural gradient in endogenous A2 concentration that went from about 1.4 μM in the T-system lumen of Endo to essentially zero in Epi, resulting in essentially 100% inhibition of I_to_ expressed in membranes of the T-system of Endo myocytes and no significant inhibition in Epi myocytes. Because of the low secretion rate, the amount of A2 diffusing out of the T-system into the vastly larger extracellular compartment was predicted to be sufficiently low to not affect systemic A2. The discovery that the system was localized to the T-system also explained a perplexing question of how A2 effects could persist in isolated myocytes, since one would think autocrine secretion into the extracellular bath would be washed away by perfusion.

The next step appears to require internalization of the activated receptors. The steady state effect of A2 on membrane transport required at least 30 minutes, a time frame that was consistent with receptor internalization and inconsistent with the initial G-protein coupled response of activated AT_1_Rs. Indeed, we found the initial G-protein coupled response of the activated AT_1_Rs was to inhibit I_CaL_ in canine ventricle [11], whereas the steady stated effect of activated receptors was to increase I_CaL_. Although these data suggested internalization was necessary, they did not rule out a role of G-protein activation in the steady state effects. However, Kim et al [12] used a “biased” agonist, that induced receptor internalization without G-protein activation, and showed inhibition of I_to,fast_ and I_K,slow_ in mouse ventricle that was identical to the steady state effects of A2.

Once the receptors are internalized, there are multiple signaling pathways that could potentially lead to the observed effects on transport. In canine left ventricle, Gao et al. [11] showed A2 coherently reduced Na/K pump current (I_p_) and transient outward potassium current (I_to_), and increased L-type calcium current (I_CaL_). In mouse ventricle, Kim et al. [12] showed A2 coherently reduced I_to,fast_ and I_K,slow._ The effect of A2 on these currents required internalization of the AT_1_Rs [11,12], so the simplest working hypothesis was that those currents that were inhibited by A2 were simply endocytosed with the receptor and were returned to the plasma membrane upon deactivation of the receptor. Since I_CaL_ was increased by A2, it would require a different signaling pathway.

Kv4.3 is the α-subunit for I_to_ in dog heart [31] and may underlie I_to,fast_ in mouse heart [32]. Regulation of Kv4.3 is the most extensively studied of the various transporters involved with the transmural gradients in transport found in canine ventricle. Rosati et al. [33] reported expression of Kv4.3 is uniform across the ventricular wall in dog, whereas the K^+^-current generated by Kv4.3 channels varies dramatically across the wall. Rosati et al did find a transmural gradient in KChip, the β-subunit for Kv4.3, and they naturally hypothesized this was responsible for the transmural gradient in current. However, based on subsequent experiments outlined below, it appears the KChip gradient is present for reasons other than regulation of K-current. Gao et al. [11] subsequently showed the transmural variation in current was dependent on A2-mediated activation of AT_1_Rs. When all AT_1_Rs were activated by 5 μM exogenous A2, the transmural gradient disappeared and I_to_ was uniformly at its minimum value whereas when AT_1_Rs were inhibited, the transmural gradient also disappeared but I_to_ was uniformly at its maximum value. This suggested existing channels were being shifted between an active and inactive state. These shifts in activity occurred in less than 2 hours, which is also more consistent with reversible post translational changes to existing proteins, rather than protein synthesis/degredation. For mouse I_to_, when AT_1_Rs were inhibited we found no difference in the maximum amplitude of either I_to,fast_ or I_K,slow_ recorded in control myocytes relative to ST-TAC myocytes or in control myocytes relative to LT-TAC myocytes. This is again consistent with the post translational hypothesis for A2-mediated regulation without changes in protein expression, even though in LT-TAC there is clearly time for changes in expression.

For Kv4.3 there is good evidence [34] it is internalized with the activated AT_1_Rs and returned to the plasma membrane when receptor activation is inhibited. Doronin et al [34] showed co-immuno precipitation of Kv4.3 with the AT_1_R in canine ventricle. They used exogenous expression of the two proteins in tissue culture and immunostaining to demonstrate Kv4.3 moved from the plasma membrane to an internal compartment upon activation of AT_1_Rs. The Na/K pump current (I_p_) in canine ventricle was inhibited with the same K_0.5_ as I_to_, so internalization with the AT_1_Rs may be the mechanism for inhibition of both currents. I_CaL_, however, is increased by internalization of the receptors, so a more intricate signaling cascade is evidently in place.

In mouse heart Kv4.3 is associated with I_to,fast_ so its inhibition may also be through internalization with AT_1_Rs. Results of the present study, however, suggest I_K,slow_ may not be part of the complex that is internalized. In LT-TAC myocytes, I_K,slow_ is uncoupled from receptor internalization whereas I_to,fast_ is not. If in ST-TAC I_K,slow_ channels are internalized with the receptor, then in LT-TAC they would have to dissociate from the receptors and relocate to a membrane domain that is not internalized with receptors. Alternatively, inhibition of I_K,slow_ may be through a different mechanism that involves reducing the open probability of the channel. In this situation, channel inhibition would require one or more steps between receptor internalization and channel inhibition. The hypertrophic program could interfere with one of these steps. While there has been significant work on signaling between AT_1_Rs and target proteins, there are still many open questions and this is an area for future investigation.

## Conclusions

This study was initiated based on the hypotheses diagrammed in Fig 1, which is largely based on work presented in Gao et al. [11]. The results presented here support some of these hypotheses, but not all, and the diagram needs to be modified.

Application of exogenous A2 to mouse myocytes [12] elicited the same reductions in I_to,fast_ and I_K,slow_ as short-term increases in load. Moreover, we showed these load-induced reductions were mediated by AT_1_R activation, which is also the path of A2-induced reductions. This is consistent with the hypothesis that autocrine secretion of A2 is stimulated by increased load to initiate electrical remodeling.

Long-term increased load induced anatomical remodeling, which added to the effect of electrical remodeling in causing increased force production. This too is consistent with Fig 1. What is not consistent is that anatomical remodeling feeds back into electrical remodeling. This could be incorporated into the diagram in general way by simply drawing an arrow from the box for anatomical remodeling to the one for electrical remodeling. This would give the general idea, but it would not tell the whole story. For the two transporters we have studied, I_to,fast_ and I_K,slow_, each is nonspecifically affected by the increased membrane area associated with hypertrophy. But the hypertrophic program specifically uncoupled I_K,slow_ from regulation by A2. Long-term load did not induce changes in the number of these two channels, but it certainly could affect synthesis and expression of other transporters. Thus the story has become much more complex and each transporter in Fig 1 may be uniquely coupled to the hypertrophic program. However, if the various interactions between the electrical and anatomical pathways can be worked out, there is potential for therapeutic benefits for persons with either low cardiac output or uncontrolled hypertrophy.

## Supporting information

S1 Fig Original ST data. (XLSX)

S2 Fig Original LT data. (XLSX)

## Abbreviations

A2: Angiotensin II
ANOVA: Analysis of Variance—statistical test
AP: Action Potential
AT_1_R: Angiotensin II Type 1 Receptor
C_m_: Cell Capacitance
Endo: Endocardium
Epi: Epicardium
HT/BW: Heart-to-Body Weight Ratio
I_Ca,L_: L-Type Ca^2+^ Current
I_K,slow_: Slow-Inactivating Transient Outward K^+^ Current in Mouse Ventricle
I_to_: Transient Outward K^+^-Current in Dog Ventricle
I_to,fast_: Fast-Inactivating Transient Outward K^+^ Current in Mouse Ventricle
LSD: Least Squares Determination—statistical test
LT: Long Term (1 to 2 Weeks)
LV: Left Ventricle
RAS: Renin Angiotensin System
RV: Right Ventricle
ST: Short Term (1 to 2 Days)
TAC: Transverse Aortic Constriction
